# Extracellular Vesicle Quantification and Characterization: Common Methods and Emerging Approaches

**DOI:** 10.3390/bioengineering6010007

**Published:** 2019-01-16

**Authors:** Thomas A. Hartjes, Serhii Mytnyk, Guido W. Jenster, Volkert van Steijn, Martin E. van Royen

**Affiliations:** 1 Department of Pathology, Erasmus Optical Imaging Centre, Erasmus MC, 3015 GE Rotterdam, The Netherlands; t.hartjes@erasmusmc.nl; 2 Department of Chemical Engineering, Delft University of Technology, 3015 CD Delft, The Netherlands; S.Mytnyk@tudelft.nl (S.M.); V.vanSteijn@tudelft.nl (V.v.S.); 3 Department of Urology, Erasmus MC, 3015 CD Rotterdam, The Netherlands; g.jenster@erasmusmc.nl

**Keywords:** Extracellular vesicles (EVs), methods for EV analysis, emerging technologies

## Abstract

Extracellular vesicles (EVs) are a family of small membrane vesicles that carry information about cells by which they are secreted. Growing interest in the role of EVs in intercellular communication, but also in using their diagnostic, prognostic and therapeutic potential in (bio) medical applications, demands for accurate assessment of their biochemical and physical properties. In this review, we provide an overview of available technologies for EV analysis by describing their working principles, assessing their utility in EV research and summarising their potential and limitations. To emphasise the innovations in EV analysis, we also highlight the unique possibilities of emerging technologies with high potential for further development.

## 1. Introduction

Extracellular vesicles (EVs) are a family of membrane vesicles containing a phospholipid bilayer and are secreted in the extracellular environment by most if not all cells. The last decade has showed a rapidly growing interest in the use of EVs in the field of biomedical research, mainly because of their high potential in clinical application and their emerging biological role in normal physiology and disease [1,2,3]. Especially EV-mediated cell-to-cell communication in cancer has been highlighted in recent years, where transfer of EVs from the tumour to the tumour-microenvironment promotes angiogenesis, matrix remodelling and modulating immune and therapy response [4,5,6,7,8]. Conversely, the transfer of EVs from the tumour microenvironment to tumour cells has been shown to promote tumorigenesis by increasing tumour cell proliferation, migration, epithelial to mesenchymal transition, and resistance to chemotherapy [7,9,10,11]. Moreover, EVs are not only transferred to neighbouring cells, but can also travel to other areas in the body where they are involved in the formation of a pre-metastatic niche [12,13,14,15,16]. 

EVs are therefore seen as a promising source for biomarkers for disease elsewhere in the body, as they reflect the cell of origin in terms of proteins, nucleic acids (mRNA and the variety of smaller non-coding RNAs) and lipids. EV-containing ‘liquid biopsies’ like blood [17], urine [18], saliva [19], and cerebrospinal fluid (CSF) [20] can be obtained in an easy and minimally-invasive way and are seen as a promising alternative to regular biopsies [21]. The biomarker potential of EVs is currently being studied in a wide variety of diseases, including cancer [18,22,23,24,25,26,27]. 

Besides their diagnostic and prognostic potential, the therapeutic use of EVs or their synthetic counterparts (e.g., liposomes) as more efficient (targeted) therapy delivery vehicles is being studied [28,29,30,31]. Human mesenchymal stem cell (MSC)-derived EVs have shown their therapeutic use in preclinical models. For example, the administration of MSC-derived EVs to mice harbouring acute kidney injury (AKI) enhanced the functional and morphologic recovery of AKI, in a comparable manner to the administration of MSCs [32]. Moreover, treatment with MSC-derived EVs reduced infarct size and local and systemic inflammation and enhanced cardiac function and geometry after myocardial ischemia/reperfusion injury [33]. The therapeutic potential of EVs is also extensively discussed within the International society for extracellular vesicles (ISEV) and the first clinical trials using EVs have been performed [34]. To utilize the biomedical potential of EVs or unravel their biological function, tools are required to determine their concentration in samples (quantification), but also to determine e.g., their size and/or molecular composition (characterization).

**Figure 1:**
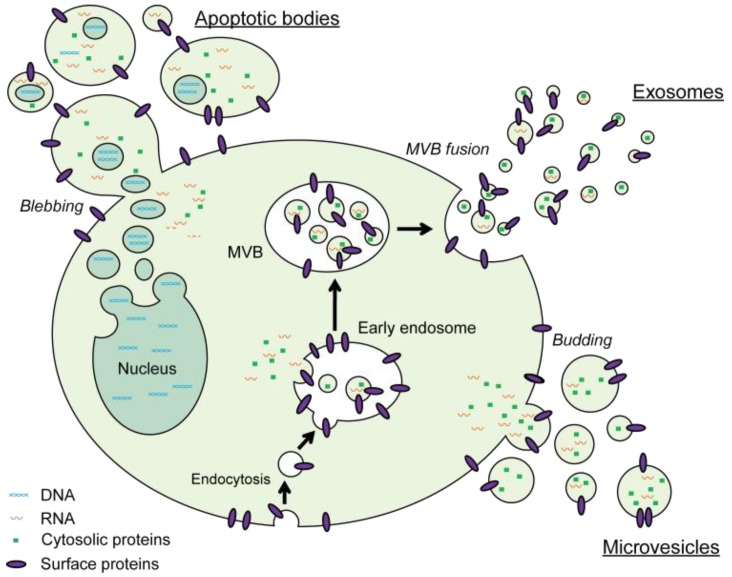
Schematic representation of the major pathways for biogenesis and secretion of extracellular vesicles (EVs). Exosomes are formed by inward budding of early endosomes and secreted by fusion of these multivesicular bodies with the plasma membrane. Microvesicles are created by direct outward budding of the plasma membrane. The vesicles that are generated upon programmed cell death-induced membrane blebbing are referred to as apoptotic bodies.

EV analysis is severely hampered by the EV heterogeneity and the complex nature of biological and clinical EV samples. The family of EVs secreted by a single cell type can be separated into three major classes based on their biogenesis: exosomes, microvesicles and apoptotic bodies (Figure 1) [35,36]. Exosomes are small vesicles with a diameter in the range between 40 to 100 nm. They are formed within endosomal compartments and secreted by the fusion of multivesicular bodies with the plasma membrane. Microvesicles are generally larger (100–1000 nm) and are formed by direct budding of the plasma membrane. Apoptotic bodies are released upon programmed cell death by membrane blebbing and can be from 50 nm up to 5 µm in diameter. However, due to a significant overlap in size, similarities in composition and lack of specific markers, it is very difficult to assign individual EVs to one of the biogenesis pathways. On top of these major classes, many specialized EV subtypes have been described [37]. To prevent any confusion about the nomenclature the ISEV community suggests in the recently updated position paper on the Minimal Information for Studies of Extracellular Vesicles (MISEV2018), to use the term “extracellular vesicle (EV)” unless the subcellular origin of the vesicle is demonstrated [38,39]. Moreover, authors are encouraged to include information on the physical characteristics of the EVs (e.g., size and/or density ranges), biochemical composition of EVs (e.g., CD63+ EVs, Annexin A5-labeled EVs) and/or descriptions of conditions or cell of origin (e.g., oncosomes, hypoxic EVs) to define EV subtypes [39].

In addition to the heterogeneity in the EV population, the presence of contaminants (e.g., protein complexes and lipoproteins) in biological and clinical EV samples should always be considered. These co-analysed or co-purified contaminants add to the challenge of EV purification, analysis and application. Therefore, proper interpretation and replication of experiments requires detailed reporting on the types of EV samples and sample handling including storage, isolation and the analytical approach used for EV analysis. While EV isolation and sample processing are very important aspects of EV research, they are out of the scope of this review. Therefore, we would like to refer the reader to excellent papers that extensively address these aspects and are meant to guide EV research and improve the quality of reported data [39,40,41,42,43].

The aim of this review is to describe the most recent advances in current technologies for EV quantification and characterization and to introduce the reader to emerging novel technologies with high potential for further development. In this fast-developing field, a variety of excellent reviews were recently published, each typically focusing on a specific EV analysis approach [44,45,46,47]. In this review, we strive to provide the reader with a broader perspective on the status of the field. First, we provide an overview of the most commonly applied methods for EV analysis together with their utility, limitations and, if applicable, important suggestions according to the MISEV initiative. We broadly classified these methods based on their detection and analysis principle into biochemical and physical analysis of EVs. In the last section, we complement this overview with a discussion on important emerging technologies that are actively being developed. By addressing the shortcomings of the commonly applied methods, these emerging technologies provide new possibilities for EV analysis and could accelerate both basic and translational EV research in the coming decades.

## 2. Common Methods for EV Quantification and Characterization

**Figure 2:**
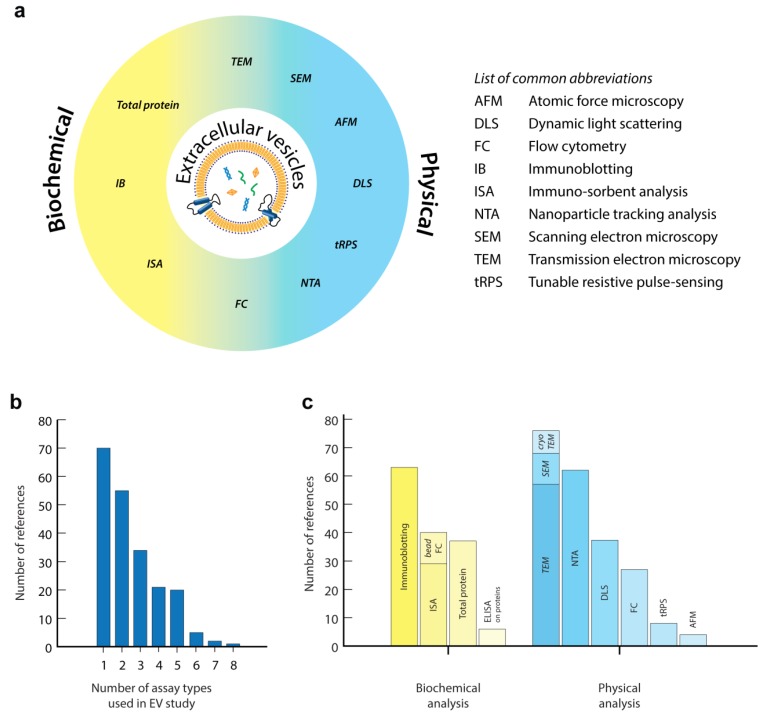
(**a**) Schematic classification of common technologies for EV analysis, as used in this review. (**b**,**c**) Analysis of the recent literature (5 years, 2013–2018) based on a PubMed search using the terms [extracellular vesicles] or [vesicles] or [exosomes] together with [quantification] in their title or abstract. From these publications, 214 available research publications described one or more technologies for EV analysis in the abstract, method or results sections (**b**). Non-English publications, reviews or publications that did not focus on EV analysis were excluded. Analysis of synthetic vesicles was included. (**c**) Frequency of usage of technologies for EV analysis in these publications, as classified as in this review. With this unbiased analysis we do not claim to cover the complete field of EV research. A more systematic analysis using targeted searches for specific techniques will certainly show additional publications on EV analysis, but this is outside the scope of the review.

The large interest in EV research combined with its challenges resulted in the development and implementation of a large variety of approaches and technologies to quantify or characterize EVs (Figure 2a). However, thus far, no single technology has shown to be able to fulfil the full spectrum of EV properties (size distribution and number of all size EVs) in complex biological or clinical samples. Moreover, the inherent heterogeneity of EVs, also reflected in a broad distribution of their biochemical and physical properties, often makes it impossible to draw concrete conclusions from bulk analysis alone. Methods that allow detection and characterization of *individual* EVs are therefore typically used in conjunction with faster, more general, *bulk* methods. Indeed, reviewing the recent literature (5 years) on the use of EV-related assays shows that the majority of EV-related studies used several complementary approaches to analyse EVs in their synthetic, biological or clinical EV samples, as is recommended by the EV-TRACK initiative (Figure 2b) [48]. The most popular approaches in recent literature are: immunoblotting of specific proteins to confirm EV origin, transmission electron microscopy (TEM) to confirm EV structure and nanoparticle tracking analysis (NTA) to quantify the number of EVs in a sample volume and their size distribution (Figure 2c). Several studies trying to unravel the biological function of EVs or focusing on biomarker discovery also used high-resolution molecular profiling of EV content (protein, RNA and lipids) using proteomics, genomics and lipidomics approaches. Although these secondary analyses cover an extremely broad field of technologies used for EV analysis, they are outside the scope of this review. We would like to refer the reader to several other excellent reviews that focus on these technologies [43,49,50,51].

This section of the review introduces the reader to general principles of the most commonly applied methods for EV analysis and summarizes their utility and limitations. To enhance readability, we broadly classified these methods based on their detection and analysis principle into biochemical and physical analysis approaches, although some techniques combine the capabilities of both classes.

### 2.1. Biochemical EV Analysis

One of the most straightforward ways of characterizing biological samples is to determine their protein composition. Also for EVs, total protein content can easily be assessed using standard colorimetric protein assays (e.g., micro-bicinchoninic acid (BCA) or Bradford assay). Although these types of assays are frequently used, they are limited to measuring highly purified EV samples since protein contaminants compromise the accuracy of the measurement. Moreover, the increased interest for EVs as a diagnostic and prognostic tool led to the use and development of biochemical analysis assays to quantify and/or identify specific EV proteins that could serve as physiological or pathological markers [52]. Here, we summarize the most commonly used biochemical methods and their recent advancements, divided into conventional protein analysis (immunoblotting assays) and assays that employ capture of (specific) EVs (immunosorbent EV assays).

#### 2.1.1. Immunoblotting

Specific proteins in EV samples are commonly detected by immunoblotting (IB). IB involves first lysing purified EVs to release their proteins, followed by either direct spotting on a membrane (in a dot blot assay), or by separation of the proteins using SDS-PAGE (in a Western blot assay) and detection using labelled antibodies targeting the protein of interest. IB is mostly used to demonstrate the presence of EV-associated proteins (e.g., CD9, CD63, ALIX, Tsg101) in order to confirm the presence of EVs in the sample [39]. According to the MISEV2018 guidelines, the presence of EVs should be demonstrated by the analysis of at least one transmembrane protein associated to the plasma membrane (e.g., CD9, CD63, CD81) and one cytosolic protein in EVs (e.g., TSG101, ALIX). Moreover, for EVs isolated from biofluids (e.g., urine, plasma), ISEV recommends additional quantification of common protein contaminants often co-isolated with EVs (e.g., apolipoproteins, albumin, uromodulin) to assess the purity of EVs [39]. Besides being a valuable tool for quality control, IB is also applicable for the detection of other (disease-) specific proteins that are present within the lumen or membrane of EVs. Even though IB enables quick and simple detection of the EV protein content, it is only semi quantitative and has the limitations of a bulk assay as it does not provide information on the protein content of individual EVs and heterogeneity within the EV population. Furthermore, this assay typically requires a large sample volume and extensive sample processing to eliminate contamination of protein sources other than the EVs. To partially address these limitations, assays that capture (specific) EVs are developed.

#### 2.1.2. Immunosorbent EV Assays

Several EV protein detection assays have been developed that use the affinity of specific antibodies for EV membrane proteins to capture and subsequently detect (specific) EVs. In these immunosorbent assays (ISAs), derived from the classical enzyme-linked immunosorbent protein assays (ELISA), EVs are typically captured on a supporting surface that is coated with an antibody targeting a common EV surface protein such as the tetraspanins CD63, CD9 or CD81 [53,54,55,56,57]. EV captures results in a strong enrichment and allows subsequent washing steps to eliminate non-EV-associated proteins that could interfere with the EV analysis. The membrane proteins of interest, present on the surface of captured EVs, are detected using antibodies targeting the same epitope of the same protein or other (disease-) specific EV surface proteins. These antibodies are directly or indirectly labelled with an enzyme like horseradish peroxidase (HRP) that induces an enzymatic conversion of a fluorescent/coloured substrate that can be quantified using a spectrophotometer (as in an ELISA). Alternatively, fluorescent antibody conjugates are used for the detection of the captured EVs in the more sensitive fluorophore-linked immunosorbent assay (FLISA) or time-resolved-fluorescence immunoassay (TR-FIA) [54,55]. Especially the prolonged (time resolved) fluorescence emission of Europium (Eu) in the TR-FIA results in minimal fluorescence levels from other sources (e.g., auto-fluorescence of the sample) and thus a superior sensitivity. These assays have shown to be valuable tools for the quantification of EV surface proteins in complex samples such as urine or blood, without prior EV isolation and/or purification [53,55,56,57]. Adding a gentle lysis step before binding of the detection antibodies allows quantification of EV cargo proteins in the TR-FIA [58].

The immunosorbent EV assays as described above can easily be carried out in a 96-well format making large scale analysis accessible and affordable. This can even be extended by miniaturization of the assay. The “ExoChip” assay, for example, makes use of a microfluidic device fabricated from silicone elastomer [59] to capture fluorescently-labelled EVs within small chambers coated with anti-CD63 antibodies, followed by EV detection using a multi-purpose plate reader. This configuration also enables recovering the EVs for further downstream analysis (e.g., RNA analysis). Assay miniaturization also allows multiplexing of EV analysis using a series of antibodies against surface targets. For example, a microarray was generated by spot-printing a panel of selected antibodies for EV capture on an epoxy-coated slide in a multi-well cassette, the “EV array” [60]. After washing away the unbound EVs, the captured ones were labelled with a cocktail of biotinylated detection antibodies against three common tetraspanins CD9, CD63 and CD81, followed by labelling with streptavidin-Cy5 for detection using a microarray reader. More recently, the authors increased the number of capture antibodies to 60 which enables in-depth surface protein profiling of EVs [61].

In addition to the classical immunosorbent approach in which a flat surface is used to capture EVs, several assays use (magnetic) beads for EV capture, e.g., the integrated microfluidic exosome analysis platform (IMEAP) [62]. The main advantage of using immuno-magnetic beads is that the capture surface is mobile which potentially increases the capture efficiency. Moreover, magnetic beads simplify labelling and washing procedures and make the assay more flexible for subsequent analyses. The use of functionalized capture beads also allows flow cytometric analysis of the bead-captured EVs using general membrane dyes and/or fluorescent antibodies [63,64]. In contrast to direct flow cytometric analysis of EVs (as discussed in the physical analysis section), this approach cannot provide information about single EV characteristics and provides only minimal insight into the heterogeneity of the EV sample. However, by using different combinations of capture antibody beads with fluorescently-labelled detection antibodies, Koliha et al. developed a multiplex bead-based platform that can detect up to 39 different surface markers in one sample and provides additional information on relative expression levels and potential EV subpopulations [65]. Using the bead immunosorbent approach, several detection variants have been developed to increase EV detection sensitivity, shorten the assay time, and make the assays more cost-effective. For example, instead of a detection antibody, a bivalent-cholesterol-labelled DNA anchor which spontaneously inserts into the EV membrane is used for detection. This DNA anchor initiates a HRP-linked hybridization chain reaction, increasing the sensitivity up to 100-fold compared to conventional ELISA [66]. Moreover, combining non-specific capture of EVs by cholesterol-modified beads and secondary labelling and fluorescent detection by copper oxide nanoparticles modified with EV-specific aptamers, shortens the assay running time from 10 h to 2 h [67]. The use of aptamers in this bead assay has a major advantage over antibodies because of low costs and their flexibility in targeting a protein of interest. A completely different use of beads is the rapid and sensitive “ExoScreen” assay [68]. In this amplified luminescent proximity assay, serum EVs are captured between two photosensitizer beads using antibodies against specific epitopes on the EV surface. Excitation of the donor bead in this sandwich conformation leads to the release of a singlet oxygen and excitation of the acceptor bead that is in close proximity, resulting in a fluorescence emission that can be detected with a plate reader. Another assay with an innovative readout is the pH-responsive assay, developed by Yang et al. [69]. EVs were captured using magnetic beads followed by secondary labelling with HRP-conjugated antibodies targeting CD63. The HRP is able to catalyse the formation of a polydopamine film on the EV surface which then can bind several ureases. The conversion of urea into ammonia and carbon dioxide by the ureases raises the pH of the solution which could be measured using commercially-available pH paper. This assay was able to reliably measure the EVs concentration down to the level of 10^6^ EVs/mL.

Despite the extensive quantitative use of immunosorbent EV assays, it must be realised that these approaches only quantify the EV-associated target proteins. Although the detection level of an EV-related protein is often used as an indication of EV concentration, observed variations in detection levels can also reflect the heterogeneous EV protein composition between biological samples, for example due to different levels of expression in the cells of origin [63,65]. Moreover, the use of specific capturing and detection antibodies implies that only a specific subset of EVs that carry the targeted proteins is being quantified and characterized. Additionally, although recent findings hint to predominant protein markers for several different EV types, no protein targets have yet been identified that cover the full spectrum of EVs or are even specifically present on every vesicle within a vesicle type [39,63]. Reporting details on the used antibodies, including source, catalogue number and concentration, is of great importance for data comparison and reproducibility [39].

The high level of standardization, the ease of use and general availability make immunosorbent EV assays very suitable for diagnostics. However, although these assays are commonly used in EV research and several immunosorbent EV assays including the TR-FIA are commercially available, their future clinical application relies on the ongoing research programs to identify essential disease markers on the EV surface.

### 2.2. Physical Analysis of EVs

In addition to determining biochemical properties of EVs using the methods described above, there is great interest to measure the concentration of EVs in a sample together with their size distribution. For example, EV size is used to infer the type of EVs (exosomes, microvesicles and apoptotic bodies), although the relation between EV size and EV type is probably less defined than suggested. It is even described that EV size and concentration vary at different stages of several types of cancer, suggesting that these parameters are potentially useful for clinical diagnostics [70,71,72].

Most common methods for size determination of EVs either determine the diameter directly, via high-resolution imaging, or indirectly, by using indirect optical or electrical readouts. Direct high-resolution imaging of immobilized EVs using EM or AFM allows for obtaining accurate size estimates of individual EVs with nanometer resolution. However, direct imaging generally does not allow for accurately establishing EV number in a given sample volume in their original state, due to the influence of complex sample preparation procedures. The methods employing indirect detection of EVs, estimate the size and/or concentration of EVs from other observable properties such as their diffusion trajectories, their interaction with light (e.g., scattering), or their effect on the electrical current within a detector. Compared to direct imaging, the number of EVs that can be analysed is typically higher for such methods, making them significantly more accurate in estimating EV concentration. Conversely, the measured size distribution is typically less accurate due to the assumptions made in translating the observed readouts to size. Additionally, indirect methods are often limited by the sensitivity of detectors or interference from other biological objects present in EV-containing samples. In this section of the review, we focus on summarizing the most relevant methods for the physical analysis of EVs by categorizing them based on the principle each of these methods use for EV size determination.

#### 2.2.1. Electron Microscopy (EM)

The most direct method to determine the size and morphology of individual EVs is electron microscopy (EM). EM employs an electron beam instead of light, which enables obtaining high-resolution images of nanoscale objects. Most commonly used types of EM are scanning and transmission electron microscopy, or SEM and TEM, respectively. In SEM, an image represents the topography of the EV surface, which is obtained by scanning it with a focused electron beam and detecting secondary electrons emitted by the atoms in the analysed area. Instead of using secondary electrons, TEM uses electrons that passed through the sample to create a 2D image of the EVs. As a result, TEM images are based on the transparency of the features of a studied object to an electron beam, offering information about the inner structure. Use of transmitted electrons limits the thickness of analysed material to 50–500 nm thin slices, depending on the power of the electron beam, making it challenging to image cells and tissues, but does not limit the analysis of EVs [73].

The main limitation of using EM on biological objects such as cells or EVs is the necessity of imaging in vacuum that generally requires fixation and drying of the sample. Such sample preparation steps complicate the translation of the observed structures to the native morphology of cells or EVs. Nevertheless, even with these limitations in mind, the size and morphology of EVs can successfully be determined using both SEM [74,75,76] and TEM [53,77]. In order to avoid sample dehydration, cryogenic EM techniques have been developed, out of which cryogenic TEM (cryo-TEM) is most suited for studying EVs. Cryo-TEM is based on imaging of ultra-thin vitrified film formed by flash-freezing thin liquid film of EV suspension at an extremely low temperature (<−100 °C) [78]. This modification of the technique allows high-resolution imaging of biological objects in their native state and is widely used to determine the ultra-structure of EVs [79,80,81,82]. Additionally, the use of immunogold labelling enables the identification of specific subsets of EVs in the presence of other similar particles in (clinical) samples [77,83]. For example, Brisson et al. combined cryo-TEM with immuno-gold labelling to study EVs derived from platelets under several types of activation and compared their size, morphology and levels of expression of CD41 and CD63 proteins [84]. Currently, cryo-TEM is regarded as one of the most reliable methods for characterization (size) of EVs. However, it offers rather limited accuracy for estimating EV concentration due to the potential influence of EV interactions with TEM grids and effects of sample blotting. The low number of EVs which is analysed by EM often makes it impossible to analyse a representative population of heterogeneous EVs (in terms of size and composition) present in biological and clinical samples. Importantly, to assess the heterogeneity in size of EVs within a sample, the MISEV initiative suggests analysing a sufficient number of overview images containing multiple EVs accompanied by close-up images of single EVs [39]. However, this hampers routine use of EM for EV analysis in a clinical setting, even if clinical relevance is proven.

#### 2.2.2. Atomic Force Microscopy

Atomic force microscopy (AFM) is a type of scanning probe microscopy that allows imaging the topology of surfaces with nanometer resolution by scanning the area with an extremely sharp tip and translating its deflection into the height of the surface features. AFM can be performed without requiring any sample labelling [85]. Most typically, AFM is used on dry immobilized EV samples, which allows estimating their size and structure. Sample damage during drying can be prevented by analysing EVs in solution, by first immobilizing them on a surface via electrostatic interactions or via binding to complementary antibodies [86,87,88]. Casado et al. used AFM to study the dynamics of EV secretion via shedding in living cells and found good correlation between the size of observed protrusions of the cell membrane and the size of EVs produced by these cells [74]. Additionally, AFM offers unique information about mechanical properties such as stiffness and elasticity of vesicles [89]. For example, the group of Wuite identified distinct differences in membrane stiffness of platelet-derived EVs between a healthy donor and a patient with hereditary spherocytosis [90]. Low throughput, requirement of specific skills and equipment are currently limiting AFM from being widely and universally applied in EV research.

#### 2.2.3. Dynamic Light Scattering

Dynamic light scattering (DLS), often also referred to as photon correlation spectroscopy (PCS), is a technique that is used to determine the size distribution of vesicles. This is done by analysing temporal intensity fluctuations of laser light, scattered by a dispersion of these freely diffusing EVs. In contrast to EM and AFM that resolve the size of individual EVs, DLS determines the collective mobility (diffusion coefficient) of scattering vesicles present in the measurement volume. The resulting size distribution is often characterized by the average size and polydispersity [91].

An advantage of DLS over other methods is the simplicity and speed of typical measurements (several minutes), making it an indispensable tool for routine EV analysis. However, due to the large number of assumptions about the nature of the particle size distribution (e.g., mono- or multi-modal) that is necessary for appropriate fitting of the measured intensity correlation function, DLS is best suited for quantitative analysis of relatively monodisperse samples. In a biological and biomedical context, it is often applied for determining the size of isolated EVs and the synthetic variants (e.g., liposomes) [92,93,94,95]. However, because DLS detects all scattering objects in solution, it has limited utility in the analysis of minimally-processed biofluids.

#### 2.2.4. Nanoparticle Tracking Analysis

The size of a particle determines how fast it diffuses in a static solution due to Brownian motion. This relation allows estimating the diffusion coefficient and size of individually observed vesicles by analysing their motion trajectories. This approach is known as single particle tracking (SPT) and forms the basis of a widely-used EV analysis method known as nanoparticle tracking analysis (NTA). Several commercial NTA operating platforms have been developed. The method is based on recording a time-lapse of particles undergoing Brownian motion by imaging them using either scattered light (Sc-NTA) or emitted fluorescence (Fl-NTA) [96]. By analysing a large number of individual trajectories, it is possible to make an estimate of the particle concentration and size distribution even in polydisperse samples.

While in principle NTA should be able to determine the size distribution of vesicles, in practice analysis is limited by the relative short measured trajectories due to continuous diffusion of vesicles in and out of focus. This results in generally high statistical uncertainties, e.g., even for 20-step trajectory expected statistical uncertainty is ~35%, leading to broadening of the obtained size distribution [97]. Statistical uncertainties can be decreased either by analysing much longer particle trajectories, which is nearly unachievable in practice, or by employing mathematical models estimating the magnitude of measurement uncertainty and correcting for it [98]. Additionally, the scattered light may misrepresent the concentration and EV size distribution in the complex biofluids due to the presence of other sources of scattering (e.g., protein aggregates) [99]. Nevertheless, even with these limitations, NTA offers fast assessment of size distribution and concentration of EVs and is extensively used in EV research. Fl-NTA is used to better distinguish EVs from other particles and by tracking only fluorescently labelled objects [96,100]. However, so far it has not become a part of standard characterization of EVs as it requires the use of very bright and photo-stable fluorescent labels (e.g., quantum dots) for detection and to avoid extensive bleaching during acquisition [100]. Finally, due to NTA being a relatively new technique it is currently undergoing active standardization which is a prerequisite for reproducibility and data interpretation [101,102].

#### 2.2.5. Tunable Resistive Pulse Sensing

Tunable resistive pulse sensing (tRPS) is a technique that detects individual nanoparticles by measuring changes in electrical current as each particle passes through an adjustable nanopore. The magnitude of the recorded drop in electrical current (blockade event) can be accurately related to the volume of the passing particle. Relatively recently, tRPS has been used to determine the size and concentration of unlabelled EVs after calibration with known standards such as polystyrene nanoparticles of well-defined size. However, while tRPS is an accurate approach, as demonstrated by its applications in nanoparticle research [103], it is still challenging to universally apply it for EV analysis of minimally-processed biological samples, mainly due to the heterogeneous nature of EV populations leading to obstruction of the pore by larger EVs and the necessity of calibration of the device in the buffer identical to the one in the sample [104,105]. Nevertheless, the availability of commercial tRPS equipment and its sensitivity make it possible to study purified EV-containing biofluids [106,107,108]. In addition to measuring EVs size and concentration, tRPS can also be used for accurate measurements of EV surface charge (zeta potential) by measuring the time each EV spends within the nanopore as a function of applied pressure and voltage [109]. This capability may find use in the design of EVs for potential therapeutic applications, since EV surface charge plays a role in their pharmacokinetic properties [110]. Taken together, the sensitivity and accuracy of tRPS make it a useful technique for studying purified EV suspensions, and further efforts in standardizing tRPS analysis protocols for better use in EV research are currently being made by several research groups [111,112].

#### 2.2.6. Flow Cytometry

Flow cytometry (FC) is commonly used for the analysis of cells and is being actively adapted for the analysis of EVs [113,114]. In FC, a flow of cells is hydro-dynamically focused in a flow chamber to enable single cell illumination by several lasers. Scattered light as a result of the difference of refractive index between cells and the solution is detected by multiple detectors. Forward scatter provides information about the size of individual cells, while side scatter provides information about their granularity and composition. Despite being a standardized and robust method for analysis of cells at a rate of a thousand cells per minute, application to EVs is a major challenge because of the low detection sensitivity for EV. Due to their small size and low refractive index difference with the solution [115,116], EVs scatter 10-fold less light compared to polystyrene beads, which are typically used for calibration [116]. As a result, most conventional flow cytometers are only able to detect single EVs above ~500 nm in size [116]. Smaller EVs are detected collectively due to so-called swarm effect [117], which occurs when multiple EVs are simultaneously illuminated by the laser. As a result, their combined scattering rises above the set detection limit, resulting in counting them as a single, much larger particle. This means that in complex samples the observed counts consist of single particle detections and swarm detections, resulting in inaccurate measurements. To estimate and possibly overcome the swarm effect, samples can be measured in serial dilutions to reach a linear correlation between the degree of dilution and the measured concentration [117]. Despite these limitations of conventional flow cytometers, FC is being used by an increasing number of research groups to study mainly larger EVs (e.g., microvesicles) [118,119]. The majority of these studies simultaneously detect scattered and fluorescent light from EVs labelled by general fluorescent membrane labels or fluorescent antibodies to enable analysis of the expression of specific surface antigens on EVs and identification of specific EV subpopulations [120,121]. However, the low number of bound fluorophores demands for highly sensitive detectors for detection of the less abundant (disease) specific EV biomarkers. This, together with the inability to detect the common small vesicles individually, severely limits the analysis of biological samples. In recent years, this clear need for more sensitive flow cytometers led to the development of dedicated flow cytometers [122,123,124,125]. Integration of a high-power 488 nm laser and special modifications of the optical detection system to decrease the forward scattering detection angle, enhanced both the scatter intensity and fluorescence signals coming from the EVs. Together with the use of immunofluorescent antibodies targeting EV-associated membrane proteins, it enabled detection and quantification of specific EV subpopulations as small as 100 nm [125]. Moreover, the introduction of a commercial imaging flow cytometer (IFC), which combines conventional flow cytometry with fluorescence imaging, enabled post analysis inspection of the detections that can be used to distinguish real EVs from protein aggregates or noise [126,127]. IFC uses a charge-coupled device (CCD) camera instead of a photomultiplier tube (PMT) which has a larger dynamic range and lower noise and is, therefore, more suitable for measuring low/weak fluorescent signals from EVs in the range of 100–200 nm [126].

Besides the need for more sensitive flow cytometers, the increasing number of EV studies using FC also emphasizes the need for standardization of sample processing and analysis for comparison of the data obtained with different instruments. Moreover, developments in calibration strategies and data processing will improve EV FC analysis. Van der Pol et al. developed a model in which the detected scattering intensity of beads with a known size and refractive index can be related to the expected size detection limit of EVs on each specific instrument. Although the refractive index of EVs in individual samples is not known, an average refractive index for EVs can be used for EV size determination based on the scatter intensity [117]. It should be noted that heterogeneity of the membrane and cargo composition of EVs affects their level of scattering and potentially introduce errors in the estimated EV sizes [128]. ISEV and several multi-center initiatives continuously improve the standardization of microparticle quantification using flow cytometry by optimizing experimental protocols, employing identical control and calibration samples, and by encouraging thorough reporting of experimental details [39,129,130]. Recent papers also extensively addressed the major challenges encountered in high-resolution flow cytometry and can serve as excellent guidelines when using this technology. This will lead to better comparability of data produced by different research groups, while minimizing the influence of previously mentioned coincidence/swarm effects [131,132,133].

Taken together, the recent advancements in the FC field, especially the ability to analyse biomarkers on individual vesicles down to 100 nm in size, will enable the identification of novel specific EV subpopulations, application of diagnostic markers on EV and might lead to deeper understanding of EV biology. However, the requirement of highly specialized and often customized equipment is a major disadvantage and makes this technique less accessible and suitable for clinical implementation. Future implementation of microfluidics in existing EV analysis techniques will allow the development of small (nano-) flow cytometers [134], capable of analysing small EVs at relatively low costs, and could well be the solution to overcome these challenges.

### 2.3. Evaluation of the Common Technologies in EV Analysis

Overall, the heterogeneity of EVs in size, composition and origin combined with the difficulty of distinguishing them from other interfering objects such as lipoproteins and viruses, in terms of size and composition, makes it challenging to standardize approaches for biochemical and physical characterization of EVs. Moreover, complex biofluids such as blood plasma contain EVs from many sources. Despite the wide variety of methods developed and implemented in EV research, each of these technologies has their potentials and limitations in the detection range, accuracy, throughput and application for the analysis of specific EV parameters (Table 1) [135]. No single technology to date is able to cover the full range of EV analyses. For example, while most physical characterization techniques are unable to differentiate EVs from different sources, the identification and use of cell type or tissue-specific capture and/or detection antibodies as in ISAs or FC, enables quantification and protein and/or genomic analysis of EVs from different origins [37]. In contrast, current biochemical analysis techniques are unable to provide information about EV heterogeneity, size and absolute concentration. Moreover, the relative quantification of EVs based on the presence of a protein of interest itself has its limitations as EVs from different origins (different cell lines, tissues or patients) greatly differ in composition due to the heterogeneous expression of proteins. This means that when the same protein levels are measured, the EV concentration can still be very different. Therefore, at this moment, the biochemical analysis is often combined with other absolute EV quantification methods to be able to normalize the obtained expression data. When it comes to physical characterization of EVs and their quantification, existing approaches are generally limited to the analysis of pre-purified EVs of a specific size range and none of the existing techniques can currently cover the full spectrum of EVs while guaranteeing the accuracy and throughput required for routine clinical analysis. Due to these reasons, ISEV recommends combining high-resolution imaging of isolated EVs using EM or AFM, together with other size and concentration measurements, such as NTA and flow cytometry [39]. Additionally, the MISEV2018 paper strongly suggests reporting all experimental details such as instrumental details (e.g., brand, software, version), settings used for acquisition (e.g., sample dilution, flow rate, camera settings, image threshold), information about controls and calibration and a precise description of the analysis procedure for every technique used for EV analysis [39]. Initiatives such as EV-TRACK have been introduced in order to promote better reporting of EV isolation, purification and analysis methodology [48]. Considering that there is a serious need in developing better tools for the analysis of EVs, the next section provides an overview of recent advances in commonly applied techniques and development of the new methods for EV characterization and quantification.

## 3. Emerging Technologies for EV Analysis

The high potential of EVs in biomedical research and the limitations of existing analytical tools have stimulated the scientific community to develop novel methods for EV characterization and quantification. In view of the previously discussed shortcomings of currently available methods, most developments focus on combining the principles of immune-labelling and immune-capture with advanced physical methods of EV detection. Some of these techniques are moving towards the characterization of individual EVs, thus offering more accurate information about EV size, concentration and protein composition, while others focus on affordable and reliable assays with the end goal of being used in a clinical setting. Notably, recent literature demonstrates a significant trend towards adaption of common techniques to a lab-on-a-chip format, thus combining the precision of original methods with very low required sample volumes and potentially much lower operational costs. Additionally, the use of the lab-on-a-chip format facilitates a range of new measurement approaches, opening new possibilities in EV research. In the remainder of this review, we summarize recent advances in EV analysis by grouping the discussed techniques based on the principle they employ for EV detection. In addition, an overview of emerging approaches, their use and additional capabilities is presented in Table 2.

### 3.1. Optical Approaches

#### 3.1.1. Fluorescence-Based Techniques

Fluorescence forms the basis of many analytical approaches in different areas of life sciences. In the context of EV research, fluorescent labelling using general lipophilic dyes or specific labelled antibodies allows visualizing and tracking them, thus enabling determination of their size and concentration. Continuous improvement of configurations of optical systems and/or illumination profiles leads to greater sensitivity and quality of EV analysis. For example, Deschout et al. recently exploited light-sheet illumination within a microfluidic device to measure the size of cell-derived EVs via fluorescence-based single particle tracking (SPT). By exciting the fluorophores only within the light-sheet, which leads to a drastic decrease in the background fluorescence of the unbound dye, the contrast greatly improves compared to epifluorescence microscopy and allows detection of vesicles with higher certainty [141]. Alternatively, the size of EVs has been studied using fluorescence correlation spectroscopy (FCS), a well-established technology that analyses temporal fluctuations of fluorescence intensity of fluorescently-labelled particles undergoing Brownian motion. Despite the fact that FCS has been previously used for the analysis of the dynamic behaviour of proteins and even for size determination of synthetic lipid vesicles, FCS has attracted the interest of EV researchers only very recently. For example, Wyss et al. recently reported an accurate analysis of highly purified EVs from cell culture using FCS with their custom algorithm for single-event analysis, which allowed measuring EVs size, concentration and the levels of CD63 on these EVs [140]. While FCS offers single fluorophore sensitivity making it a promising tool in EV research, it still requires further standardization and validation for analysis of EV-containing samples. Very recently, a fluorescent microscopy-based single EV analysis (SEA) technique was developed allowing multiplexed biomarker analysis of individual EVs. EVs are immobilized in a microfluidic device followed by on-chip immuno-staining (up to three markers) and acquisition of fluorescent images. By quenching the fluorophores already present on EVs followed by labelling with three additional detection antibodies and subsequently repeating, this procedure makes it possible to detect more than 10 specific markers on the same EVs [139].

#### 3.1.2. Surface Plasmon Resonance (SPR)

Several emerging technologies for EV analysis are based on surface plasmon resonance (SPR). SPR enables highly sensitive label-free detection of EVs by their immune capture to an SPR-active surface such as gold or silver nanoparticles. Some of these techniques have been recently employed for quantifying tumour-derived EVs based on selected protein markers. For example, gold nanoparticles stabilized with DNA aptamers against specific surface proteins have been shown to produce a clear colour change due to the specific binding of EVs to these aptamers [144]. This approach allows for analysing EVs protein content both visually and spectrophotometrically in a multiplexed approach. The shape of surface used is highly variable and can be adapted to the assay format. Im et al. developed a microfluidic SPR platform that uses changes in transparency of a thin gold layer with nanoholes triggered by immune capture of EVs for determining the concentration of EVs expressing several common protein markers [143]. Alternatively, single capture events of EVs derived from a purified breast cancer cell line were detected by using localized SPR imaging (LSPRi) of a nano-fabricated gold nanopillar array modified with anti-CD63-antibodies [142]. Lastly, self-assembled gold nano islands on glass were immuno-modified for measuring the concentration of EVs derived from a series of cultured cell types [145].

Over the last decade, another SPR-based technique, surface-enhanced Raman spectroscopy (SERS), started playing an important role in the biochemical analysis of low-abundance biomarkers. The signal enhancement through the SERS effect, allows detection of single molecules captured on antibody-modified metal nanoparticles. However, only very recently, several studies have described SERS-based assays and devices for measuring concentration and protein profiling of EVs [146,147,148,149,150,151]. The clinical relevance is even suggested by differences found in Raman spectra of dried EVs derived from lung cancer cells versus normal alveolar EVs using SERS [146]. Several sandwich-type SERS immunoassays have been reported, which first concentrate EVs using immuno-capture with magnetic beads, and then detect captured EVs using immuno-labelled SERS-nanoprobes. This strategy allowed researchers to develop multiplexed assays with sensitivity to EV concentration down to ~4 × 10^4^ mL^−1^ [147,148,149]. Gold nanorods were used as SERS nanotags to count plasma EVs and determine their level of even eight common protein biomarkers in a glass-slide-based assay [150]. By combining dielectrophoretic trapping of model vesicles with SERS imaging, Ertsgaard et al. measured the Raman spectra of EV contents, thus presenting a new tool for high speed analysis of composition of EVs [151]. A relatively low-cost alternative to the currently employed gold nanoparticles as SERS surface is the use of CD-R discs as a template for a silver nanolayer. This assay is applied to measure Raman spectra of haemoglobin and plasma EVs [163].

#### 3.1.3. Interferometric Imaging

Digital optical detection and counting of individual EVs is currently gaining popularity, and one such technique, reported by Daaboul and colleagues, is based on interferometric imaging of EVs captured on a layered silicon substrate, allowing EV size to be related to the contrast of observed bound vesicles [152]. This platform, called Single Particle Interferometric Reflectance Imaging Sensor (SP-IRIS), allows multiplexing the analysis by creating an array of different immobilized antibodies, and so far it has been demonstrated in simultaneous sizing and protein profiling of purified EVs from human cerebrospinal fluid using the abundant CD9, CD63 and CD81 markers. The authors claim that the method is sensitive enough to even detect EVs of 40 nm. This technology is currently being developed into a cartridge-based platform under the name “ExoView”.

### 3.2. Electrochemical Sensing

A relatively new field for EV quantification and characterization is electrochemical analysis, with several techniques recently being developed. The main advantage of the electrochemical assays compared to standard immunosorbent assays is their very low detection limits. This allows the use of small sample volumes or detects low EV concentrations in strongly diluted samples, reducing the effect of contaminants (i.e., protein complexes, lipoproteins) during the analysis of complex clinical samples such as blood plasma. The electrochemical biosensors can easily be miniaturized and have high potential for implementation into portable devices which could accelerate the transition to point-of-care diagnostics.

To decrease the detection sensitivity down to hundreds of EVs per millilitre, innovative capture and detection strategies were developed that enabled EV detection by an electrochemical readout. Wang et al. developed novel nanotetrahedron aptamers for capturing EVs on gold electrodes, which in combination with electrochemical readout, resulted in 100-fold increased EV detection sensitivity compared to single-stranded aptamer sensors [153]. EVs from breast cancer cells with a concentration down to 10^2^ EVs/mL could be detected without the use of labels by monitoring the changes in electrochemical parameters of the system due to binding of CD81-containing EVs to immuno-modified gold electrodes via differential pulse voltammetry (DPV) and via electrochemical impedance spectroscopy (EIS) [154]. The use of quantum dots enabled signal enhancement in anodic stripping voltammetry quantification for the highly sensitive detection of the less abundant disease-specific markers for colorectal adenocarcinoma on tumour-derived EVs in serum to a concentration of 10^5^ EVs/mL [155]. Alternatively, an ELISA-based assay uses a gold substrate coated with EV capture antibodies targeting CD9 and EV detection using HRP-mediated electrochemical reduction of an indicator monitored by amperometric readout [157]. In contrast to most assays that focus on quantification or characterization of EV membrane proteins, Tu et al. developed an assay called electric field-induced release and measurement “EFIRM”, which is able to quantify EV cargo [156]. After EV capture using anti-CD63 antibody-labelled magnetic beads, the membrane of the EVs is disrupted by low-voltage electric cyclic square waves (CSW), leading to the release of EV cargo. Specific RNAs or proteins released from the EVs are then hybridized to DNA primers or antibodies on an electrode surface followed by quantifying of the captured proteins or RNAs based on the changes in electrical current. This assay has the unique capability to quantify EV cargo without using chemical lysis, which might interfere with down-stream analysis procedures.

### 3.3. Filter Paper-Based Techniques

Several research groups are exploring the possibilities of paper-based detection of EVs. These procedures are simple, fast, low-cost, and widely available, making them a perfect candidate for clinical implementation and point-of-care analysis. For example, Chen et al. developed a paper-supported aptasensor based on luminescence resonance energy transfer (LRET) from upconversion nanoparticles to gold nanorods [159]. A DNA aptamer of the CD63 protein is split in two and one part is immobilized on a filter paper surface together with the upconversion nanoparticles, while the other part is attached to the gold nanorods and added together with the EV sample on the paper. The CD63 protein on the exosomes binds to both parts of the aptamer which minimizes the distance between the upconversion nanoparticles and the gold nanorods inducing LRET. The decrease in green luminescence is recorded by a CCD camera and a relationship between the quenching rate and concentration of EVs was calculated. The high sensitivity (LOD of 10^6^ EVs/mL) and the more important short assay time (30 min) make this assay a very interesting candidate for point-of-care analysis. With a completely different approach, Oliveira-Rodriguez et al. developed a rapid lateral flow immunoassay in which a nitrocellulose membrane containing a lane of anti-CD9/CD81 antibodies for EV capture is shortly incubated in a well containing a mix of EVs and anti-CD63- conjugated gold nanoparticles [158]. The EV-gold nanoparticle complexes will bind to the test lane, but unbound gold nanoparticles will continue to flow to the end of the membrane. The EV-gold nanoparticle complexes will bind to the test lane, while unbound gold nanoparticles will continue to flow to the end of the membrane. The high concentration of bound nanoparticles present in the test lane results in the appearance of a coloured band easily detected by eye which could be beneficial for the development of a home test. Image analysis of the coloured bands showed that this assay has a limit of detection of 10^9^ EVs/mL.

### 3.4. Other Techniques

In addition to techniques discussed in the previous sections, there have been several reports of innovative methods for EV detection. For instance, a microfluidic assay has been developed to measure the concentration of the EVs via nuclear magnetic resonance (NMR) detection of vesicles containing various glioblastoma biomarkers by binding them to anti-CD63-modified magnetic nanoparticles [162]. Another innovative approach to measure EV size was recently reported by Oclum and co-workers, who used the change in resonance frequency of AFM cantilever containing a nanochannel to determine the buoyant weight of nanoparticles and EVs flowing through it [161]. The suspended nanochannel resonators (SNRs) allow precisely measuring the weight of nanoparticles down to 5 attograms, which is approximately equivalent to the weight of 40 nm EVs, with a throughput of several thousands of particles per hour. Using this technique, the authors have measured weight distributions of EVs derived from two different cell lines. Furthermore, size exclusion chromatography (SEC) has been demonstrated not only for EVs isolation and purification, but also for their quantification. Xu et al. developed a SEC assay with fluorescence detection (SEC-FD), which allows separating fluorescently-labelled EVs from other components of the medium and quantifying EV concentration based on fluorescence intensity of the eluted fraction [160]. They have applied SEC-FD for monitoring the production of EVs by a human cell line, successfully showing the detection of EVs at concentrations >3 × 10^7^ particles/mL, which was sensitive enough to use for the analysis of small volumes (0.5 mL) of cell culture medium without enrichment.

## 4. Conclusions and Outlook

Modern EV research relies on a wide range of biochemical and physical analysis approaches. Even though some of the challenges in EV analysis remain unsolved, the rapid development of a large variety of advanced assays for EV analysis has led to major improvements in the quantification and characterization of EVs. While most methods provide information about EV populations as a whole, there is great interest in complementing these techniques with methods that are able to determine parameters, including size and molecular content at the single vesicle level. Moreover, it has become clear that the analysis of individual EVs and the identification of (clinically) relevant subpopulations is essential for the proper interpretation of EV-related studies and could well provide new opportunities in biomarker research and their application in diagnostic assays. The increased interest for EVs in biomedical research and the importance of the ongoing collaborations with applied biophysics and chemistry is illustrated by the growing number of advanced EV characterization approaches being developed, often based on detection principles previously applied in these research fields.

Furthermore, rapid development of microfluidic and lab-on-a-chip technologies enabled miniaturization of many techniques, integrating EV isolation, purification and analysis within a single assay. These developments have also led to a significant decrease in sample volume necessary for analysis, as is often required for biomedical and clinical application. Future advancements in this field will certainly lead to technology for fast and reliable EV quantification and characterization assays at low costs. Furthermore, the active and continuously growing EV research community and our increasing understanding of challenges and pitfalls, will lead to improvements in the reliability and reproducibility of reported data.

## Figures and Tables

**Table 1:** Summary of the capabilities of the most common methods for EV analysis. Methods are grouped according to their type (biochemical or physical analysis), with subgroups based on the principle of EV detection. Evaluation of performance of each technique is given by the number of stars, with * being the poorest performance and *** being best performance out of the compared techniques.

Technique	Detectable Size Range	Measurement Type	Accuracy EV Concentration	Sample Processing	Measurement Time	Size Distribution	Protein Profiling	Additional Capabilities	Further Information
**Biochemical Analysis**									
Total protein content	−	Bulk	**	***	***	−	−	-	[136]
Immunoblotting	−	Bulk	*	*	*	−	+	-	[115,137]
Immunosorbent assays	−	Bulk	*	***	*	−	+	96-wells format	[55,60,62]
**Physical Analysis**									
*Direct imaging*									
EM	>5 nm	Individual	*	*	*	***	+	Cryo-TEM for imaging hydrated EV; immunogold labelling for phenotyping	[73,77]
AFM	>5 nm	Individual	*	*	*	**	−	Mechanical properties of EV membranes	[85]
*Indirect optical detection*									
DLS	5–2000 nm	Bulk	**	***	***	**	−	Surface zeta potential measurement	[91,138]
NTA	50–1000 nm	Individual	**	**	**	**	+/−	Immunofluorescent labelling	[100,101]
Flow cytometry									
*Scattering*	>300 nm	Individual	**	***	**	*	−	-	[114,130]
*Fluorescence*	>100 nm	Individual	***	**	**	*	+	Immunofluorescent labelling	[121]
*Indirect non-optical detection*									
TRPS	>30 nm	Individual	***	*	**	**	−	Surface zeta potential measurement	[104,109]

**Table 2:** Summary of the properties of emerging approaches for EV analysis categorized by the main principle of EV detection.

Assays	Measurement Type	Size Measurement	Additional Capabilities	Used Sample Type	Further Information
*Fluorescence-based techniques*					
Single EV analysis (SEA)	Individual	−	Multiplexed immunoassay	Cell medium	[139]
Fluorescence correlation spectroscopy (FCS)	Bulk	+	Simultaneous size and concentration measurement	Cell medium	[140]
On-chip light sheet illumination	Individual	+	Simultaneous size and concentration measurement	Cell medium, Interstitial fluid	[141]
*Surface plasmon resonance (SPR)*					
Classic SPR sensors; Localized SPR imaging (LSPRi)	Bulk Individual	−	High sensitivity Label-free detection	Cell medium, Blood, Urine	[142,143,144,145]
Surface-enhanced Raman spectroscopy (SERS)	Bulk	−	Molecular composition	Cell medium, Blood	[146,147,148,149,150,151]
*Interferometric imaging*					
ExoView	Individual	+	Multiplexed immunoassay	Cell medium, CSF	[152]
*Electrochemical sensing*					
Nanotetrahedron-assisted electrochemical aptasensor	Bulk	−	High sensitivity Low cost	Cell medium	[153]
Differential pulse voltammetry (DPV) and impedance spectroscopy (EIS)	Bulk	−	High sensitivity Low cost	Cell medium	[154]
Quantum dot-based enhanced stripping voltammetry	Bulk	−	High sensitivity Multiplexing (unpublished)	Cell medium, Blood	[155]
Electric field-induced release and measurement (EFIRM)	Bulk	−	Quantification of EV cargo proteins/RNAs	Blood, Saliva	[156]
Amperometric biosensor based on surface marker-mediated signal amplification	Bulk	−	High sensitivity/specificity	Cell medium	[157]
*Filter paper-based immunoassays*					
Lateral flow immunoassay (LFIA)	Bulk	−	Low cost Minimal processing	Cell medium, Blood, Urine	[158]
Aptasensor based on luminescence resonance energy transfer (LRET)	Bulk	−	High sensitivity Low cost	Cell medium	[159]
*Other techniques*					
Size-exclusion chromatography (SEC)	Bulk	−	Simultaneous purification	Cell medium	[160]
Suspended nanochannel resonators (SNRs)	Individual	+	Weight estimate of individual EVs	Cell medium	[161]
Micro nuclear magnetic resonance	Bulk	−	High sensitivity	Cell medium, Blood	[162]
